# A Novel Approach to Increase the Oxygen Permeability of Soft Contact Lenses by Incorporating Silica Sol

**DOI:** 10.3390/polym12092087

**Published:** 2020-09-14

**Authors:** Nguyen-Phuong-Dung Tran, Chuan-Cheng Ting, Chien-Hong Lin, Ming-Chien Yang

**Affiliations:** Department of Materials Science and Engineering, National Taiwan University of Science and Technology, Taipei 10607, Taiwan; thaonguyeng89@gmail.com (N.-P.-D.T.); jeffting2926198619@gmail.com (C.-C.T.); chlin0805@gmail.com (C.-H.L.)

**Keywords:** silica sol, soft contact lens, poly(HEMA-*co*-NVP), oxygen permeability, hydrophilicity

## Abstract

This study presents a novel approach to increase the oxygen permeability of hydrogel by the addition of silica sol. Herein, 2-hydroxyethyl methacrylate (HEMA) was copolymerized with N-vinyl-2-pyrrolidone (NVP) after mixing with silica sol. The resultant hydrogel was subject to characterizations including Fourier-transform infrared (FTIR), equilibrium water content (EWC), contact angle, optical transmittance, oxygen permeability (Dk), tensile test, anti-deposition of proteins, and cytotoxicity. The results showed that with the increase of silica content, the Dk values and Young’s moduli increased, the optical transmittance decreased slightly, whereas the EWC and contact angle, and protein deposition were not much affected. Moreover, the cytotoxicity of the resultant poly(HEMA-*co*-NVP)-SNPs indicated that the presence of silica sol was non-toxic and caused no effect to the growth of L929 cells. Thus, this approach increased the Dk of soft contact lenses without affecting their hydrophilicity.

## 1. Introduction

Currently, contact lenses are manufactured from hydrogels of hydrophilic monomers such as 2-hydroxyethylmethacrylate (HEMA) and 1-vinyl-2-pyrrolidinone (NVP). Hydrogel lenses were required to supply sufficient oxygen to the eyes during walking and sleeping. In general, for soft contact lenses, there are two possible avenues to increase oxygen permeability: develop the materials with higher water content or develop the hydrophobic materials. Oxygen can be absorbed and permeate through the soft lens by water phase or its matrix [1,2]. In conventional hydrogel, oxygen permeability is essentially governed by the equilibrium water content (EWC). Higher water content in soft lens can achieve higher oxygen transmissibility. However, the permeability of water (Dk_water_) was approximate 80 barrer, restricting the delivery of oxygen to the eyes through hydrogel lenses [1]. In addition, high water content in hydrogel matrix may reduce the mechanical properties and increase protein deposition of contact lenses [3]. In the other permeation path based on the matrix, hydrophobic silicon-containing ingredients, such as polydimethylsiloxane (PDMS) and 3-(methacryloyloxy) propyltris(trimethylsiloxy) silane (TRIS), are incorporated into hydrogels to deliver sufficient oxygen to the eyes. Combining hydrophilic and hydrophobic components often incurs phase separation that impairs the transparency of the lens. Feasible approaches were developed in the manufacturing of commercial silicone hydrogel lenses [4,5]. Other than the lens’s transparency, silicone materials exhibit some problems because of their characteristics. High oxygen supply with high silicone content is frequently in the expense of water absorbability as well as wetting surface [3,6,7,8,9]. High silicone content may also reduce resistance to protein deposition and mechanical properties [3,6,10,11,12].

A new approach to solve problems associated with silicone and to achieve high water content may be developed based on tetraethyl orthosilicate (TEOS) via Stöber process. Silica nanoparticles (SNPs) synthesized from TEOS were reported when blending into polymers could increase the gas permeability [13,14]. The oxygen permeability was enhanced to about 40% higher than the theoretical prediction. Based on this finding, we proposed a novel approach to increase the oxygen permeability by blending silica nanoparticles while keeping the hydrophilicity of the base hydrogels. In this study, SNPs were synthesized from TEOS through sol-gel process before mixing with the matrix of hydrophilic materials such as HEMA and NVP. All the resultant SNPs-loaded poly(HEMA-*co*-NVP) were characterized by dynamic light scattering (DLS), Fourier-transform infrared spectrometer (FTIR), equilibrium water content (EWC), oxygen permeability (Dk), contact angle, optical transparency, mechanical properties, protein adsorption, and in vitro cytotoxicity.

## 2. Materials and Methods

### 2.1. Materials

Tetraethyl orthosilicate (TEOS), 1-vinyl-2-pyrrolidinone (NVP), phosphate buffered saline powder (PBS, 0.1 M, pH 7.4), and 2-hydroxy-2-methylbenzene acetone (D-1173) were purchased from Sigma-Aldrich, St. Louis, MO, USA. Glycerol, 2-hydroxyethylmethacrylate (HEMA), and ethylene glycol dimethacrylate (EGDMA) were obtained from Acros Organics (Morris Plains, NJ, USA). Phosphate buffered saline solution (1 wt%, pH 7.4) was prepared in our laboratory.

### 2.2. Preparation of Silica Nanoparticles and Hydrogels 

**Figure 1:**
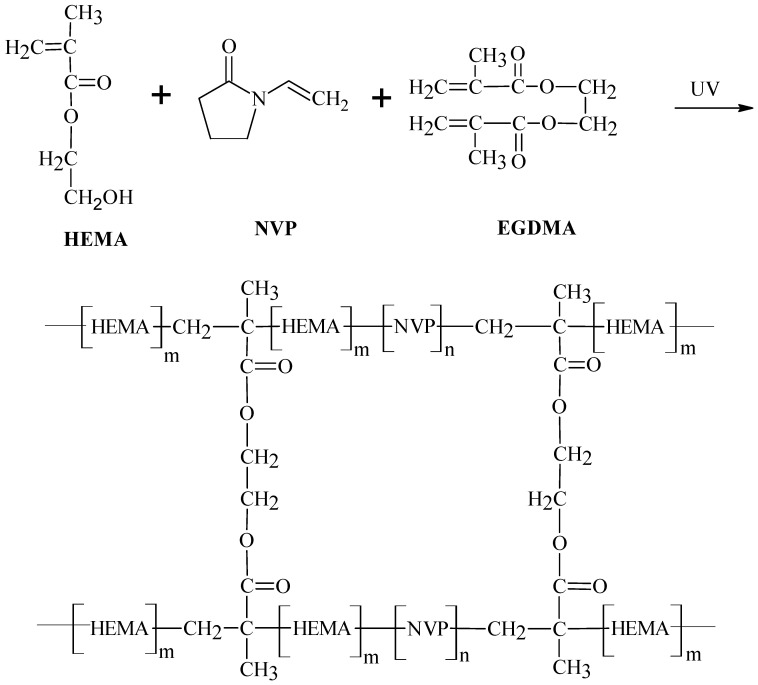
The reaction of poly(HEMA-*co*-NVP) lenses.

Silica nanoparticles (SNPs) were prepared through the sol-gel process. Briefly, 1 g of TEOS was slowly added into 5 mL of 0.5 M HCl at room temperature and stirred for 24 h. Afterward, the size of the SNPs in the sol was analyzed through dynamic light scattering (DLS-DKSH, Nano-ZS90, Malvern Instruments Ltd., UK). After verifying the synthesis of SNPs, an aliquot of the silica sol, ranging from 0 to 2 g, was added to 4 g of HEMA followed by 1 g of NVP, 0.5 g of glycerol, 0.1 g of EDGMA (crosslinking agent), and 0.1 g of D-1173 (photo-initiator). The solution was mixed for 1 h and molded before exposing to UV light (365 nm) at 5 mW/cm^2^ for 20 min. After demolding, the resultant lenses were soaked in 50% ethanol for 20 h at 50 °C to remove un-reacted monomers and photo initiator. Then, the lenses were immersed in distilled water for 4 h at 50 °C to wash out ethanol. Finally, the lenses were preserved in PBS (pH 7.4) at room temperature. Table 1 presents the compositions of the samples. Figure 1 shows the synthesis of the hydrogels.

### 2.3. Characterization of SNPs and Hydrogel Lenses

#### 2.3.1. Size of Particles and Chemical Structure

The size of the SNPs in the sol was analyzed through dynamic light scattering (DLS-DKSH, Malvern Instruments Ltd., Malvern, UK). The chemical structure of SNPs was examined using Raman Spectroscopy (iHR550, Horiba Scientific, Kyoto, Japan) via the wavenumber range of 400–4000 cm^−1^.

#### 2.3.2. Fourier-Transform Infrared Spectrometer

The structure of the hydrogels was characterized in the wavenumber range of 600–4000 cm^−1^ by using a Fourier-transform infrared spectrometer (FTIR, Nicolet 170 SX, Thermo Fisher Scientific, Madison, WI, USA).

#### 2.3.3. Equilibrium Water Content

Equilibrium water content (EWC) of lenses was calculated as follows:

(1) $$\mathrm{EWC}\;(\%)=\frac{W_{w}-W_{d}}{W_{w}}\times 100$$

where W_d_ and W_w_ are, respectively, the weights of the dry and rehydrated samples. The rehydrated lenses were immersed in distilled water for one day at room temperature before being weighed.

#### 2.3.4. Oxygen Permeability

The oxygen permeability (Dk) of poly(HEMA-*co*-NVP)-SiO_2_ was determined using an oxygen permeometer (Model 201T, Createch, CA, USA) based on polarographic method.

#### 2.3.5. Contact Angle

The contact angle was determined using a contact angle goniometer (DSA 100, Krüss GmbH, Hamburg, Germany) at 37 °C.

#### 2.3.6. Optical Transparency

The optical transparency of the contact lens was measured based on light transmittance (T%) in a wavelength range of 400–700 nm using a UV-Vis spectrometer (Cary 300, Agilient Technologies, Santa Clara, CA, USA).

#### 2.3.7. Mechanical Properties

The mechanical properties of all specimens were measured using a tensile tester (MTS 810, Material Test System, MN, USA). The hydrated specimens were cut into dog bone shape before measuring the modulus at a crosshead speed of 50 mm/min.

#### 2.3.8. Protein Deposition

Protein deposition on the contact lenses was studied against human serum albumin (HSA) and lysozyme using a UV-Vis spectrometer. Specimens (1 × 1 cm) were immersed in 1 mL PBS containing 2 mg/mL of either HSA or lysozyme. After being incubated at 37 °C for 24 h, specimens were rinsed 3 times with PBS. Then, specimens were placed into 2 mL of 1 wt% sodium dodecyl sulfate (SDS), and shaken at 100 rpm at 37 °C for 1 h to remove the protein adsorbed on the lenses surface. Finally, the protein deposition on the lenses were analyzed based on BCA assay after being incubated in 2 mL of bicinchoninic acid at 37 °C for 1 h.

#### 2.3.9. Cytotoxicity Test

The cytotoxicity test of the soft lenses was analyzed according to ISO 10993-5. The cell culture medium for determining cytotoxicity was a mixture of 94% Dulbecco’s Modified Eagle’s medium (DMEM), 1% penicillin antibiotic (PNC), and 5% fetal bovine serum (FBS). After being sterilized under the UV light for 4 h, specimens (1 × 1 cm) were soaked in cell culture media for 24 h at 37 °C. Then, the extracted media were filtered by passing through a 0.22 µm filter. L929 cells were cultured with the extracted media, positive control, and negative control. Thiazolyl blue tetrazolium bromide (MTT) reagent was mixed into the cells at 37 °C for 4 h after being incubated for 48 h at 37 °C in a humidified atmosphere containing 5% carbon dioxide. Afterwards, dimethyl sulfoxide (DMSO) was added into the media to dissolve the purple product. Finally, the cytotoxicity test was analyzed at 570 nm using an Elisa reader (M965, Accu reader, Taiwan).

## 3. Results

### 3.1. Dynamic Light Scattering and Raman 

**Figure 2:**
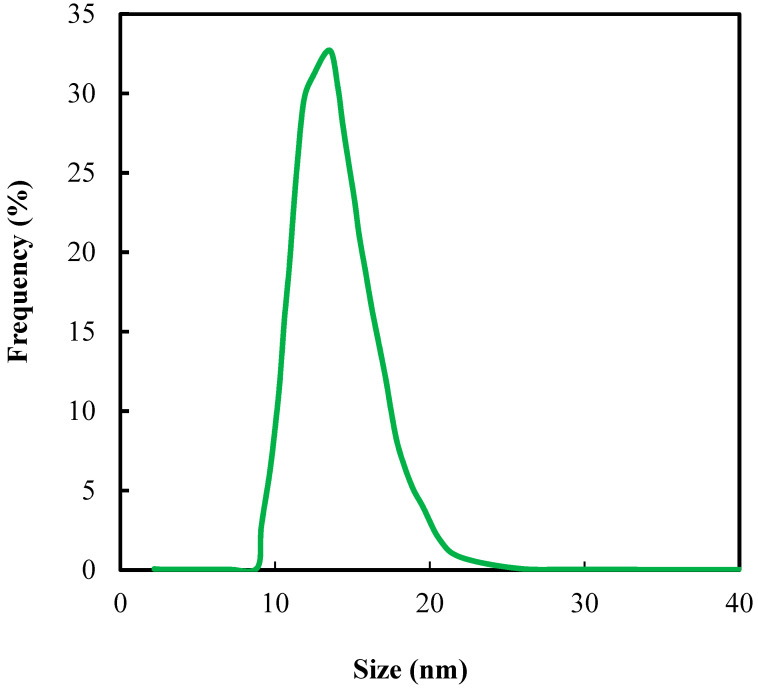
DLS of SNPs.

Figure 2 shows that the mean diameter of the resultant SNPs was 13.5 nm determined by DLS measurement. In general, the hydrolysis and condensation of TEOS in water would first result in a sol, which is composed of SiO_2_ nanoparticles suspending in water. After a period of time, these nanoparticles would further condense to become gel. In this present study, the condensation was terminated before the gelation by adding the sol directly to the monomer solution. Glycerol was added to improve the miscibility of the silica sol and the monomers. The solution was polymerized under UV (365 nm) followed by rinsing with ethanol and water to remove glycerol and unreacted monomers.

**Figure 3:**
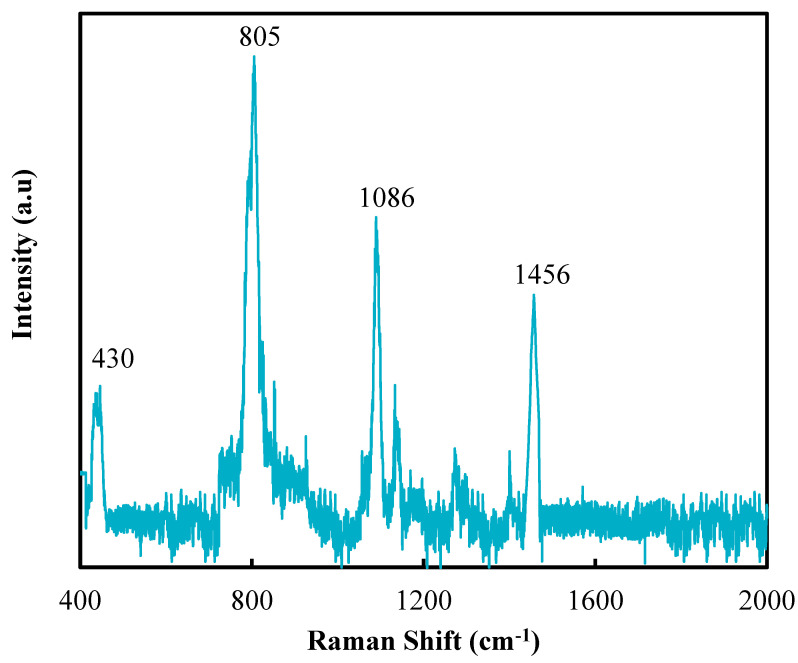
Raman spectrum of SNPs.

Figure 3 presents the Raman spectrum of SNPs through the sol-gel process. The peak of Si–O group was detected at 805 cm^−1^ while the peaks of C_2_H_5_OH were respectively observed at 430 cm^−1^ and 1086 cm^−1^ (C–O groups) and at 1456 cm^−1^ (CH_2_ groups).

### 3.2. Fourier-Transform Infrared Spectrometer

**Figure 4:**
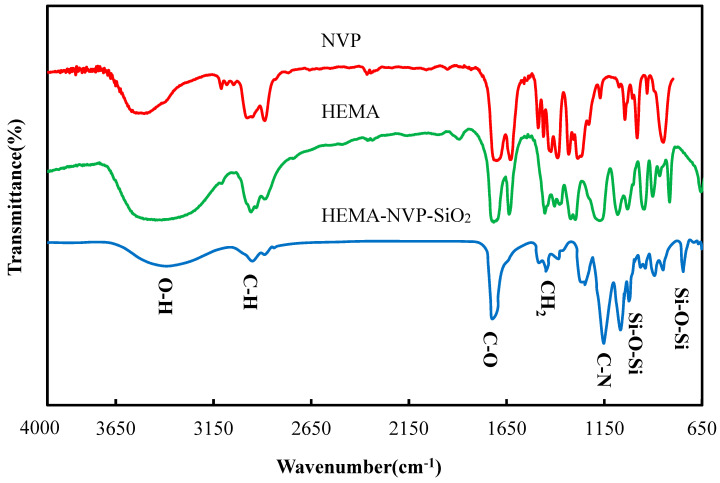
FTIR spectra of SNPs lenses.

Figure 4 shows the FTIR spectra of HEMA, NVP, and poly(HEMA-*co*-NVP)-SNPs, where the peaks of C–H groups (2878 cm^−1^) and C=O groups (1720 cm^−1^) were observed. Additionally, in SNP-loaded lenses, the peaks of Si–O–Si group in SiO_2_ were appeared at 720 and 1068 cm^−1^ while the C–N group of NVP were observed in 1150 cm^−1^ [6,15]. The characteristic peaks of C–C double bond were absent from the infrared spectrum of the resultant lens, indicating the successful synthesis of the hydrogel.

### 3.3. Equilibrium Water Content

Table 1 shows that the equilibrium water content (EWC) of SNPs lenses was influenced by loading of SNPs various concentrations. The EWC value of lenses varied slightly around 52%, regardless of the content of SNPs. The quantity of nanoparticles added was small, and that SNPs had little interaction with the matrix of HEMA and NVP Thus EWC was maintained for the hydrogel of hydrophilic HEMA and NVP [3,16].

### 3.4. Contact Angle

Table 1 presents that contact angle was basically independent on the SNP content. The contact angle was varied around 60° while the SNPs concentration increased from 0 wt% to 1.8 wt%. The possible reason was that the SNPs exhibit content was less than 1.8%hydroxyl groups on the surface, making them hydrophilic and was embedded in the hydrogel matrix, thus affected little the hydrophilicity wettability of the hydrogel. This result also has the same tendency as stated in literature. The combination of small amount of SiNPs silicone nanoparticlescontent with hydrophilic monomers had the slight influence on the wetting surface of soft lenses [17].

### 3.5. Optical Transparency

**Figure 5:**
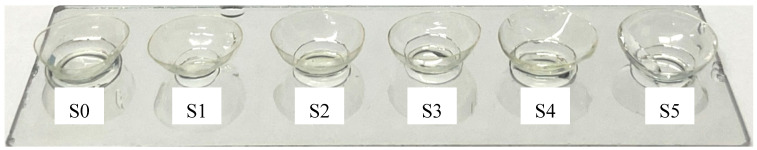
Photos of SNPs hydrated contact lenses.

**Figure 6:**
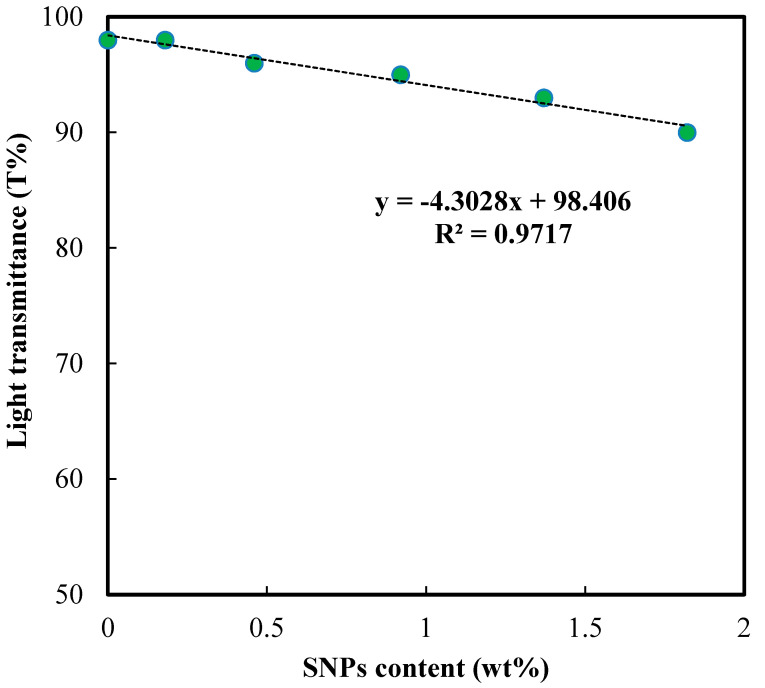
Effect of SNPs content on optical transparency.

Figure 5 presents the photos of SNPs hydrated contact lenses. Figure 6 shows that the light transmittance decreased with the increase of SNP content. The transparency of commercial contact lenses is usually above 90% [3,17], thus in this work, the maximum SNP content was limited to 1.8 wt%. The decrease in the light transmittance is probably caused by the formation of nodules due to the interaction between SNPs and functional groups in poly(HEMA-*co*-NVP), which scattered the light. A higher SNP content leads to more nodules, and thus impairs light transmission.

### 3.6. Oxygen Permeability

**Figure 7:**
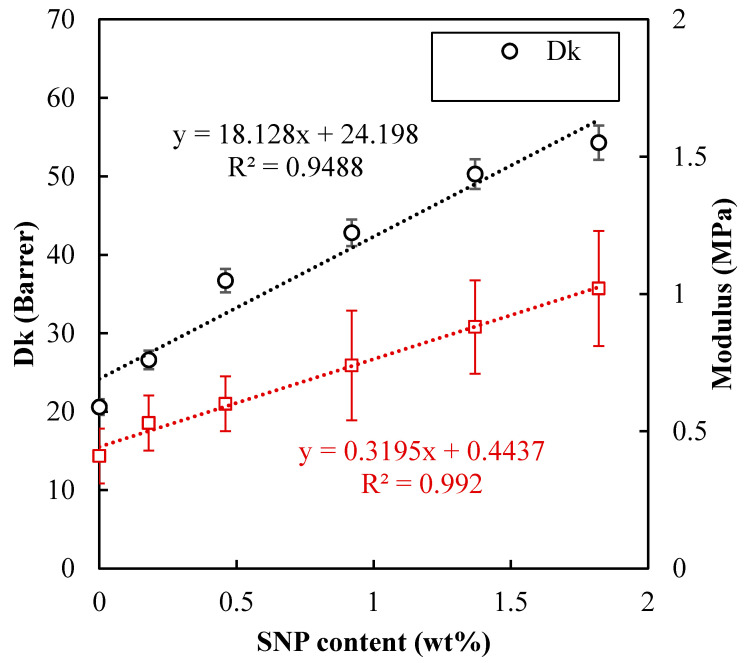
Effect of SNPs content on oxygen permeability and Young’ modulus.

Increasing Dk for hydrogel lenses using hydrophobic groups is important to restrict the corneal damage such as hypoxia, corneal edema, and red eyes [18,19]. Figure 7 shows that the oxygen permeability of SNP-loaded hydrogel increased with the increase of SNP content. The highest Dk reached at 54.3 barrer corresponding to the highest SNPs ratio (1.8 wt%). This trend is quite unusually because SiO_2_ exhibits an oxygen permeability much lower than polymers and water. The presence of silica should hinder the diffusion of oxygen through the hydrogel. However, in SNPs-loaded hydrogel, these particles were entrapped in a hydrated environment. This would confer SNPs mobility, facilitating the creation of free volumes for oxygen to pass through, thus accelerating the diffusion of oxygen. This finding would be useful for developing novel materials for contact lens.

In general, the inverted correlation frequently appears between Dk and EWC in soft lenses whereby the increase of hydrophobic ingredients such as PDMS and TRIS will affect the water absorbability of soft lenses as reported previously [7,20,21]. However, in this study, the inverted relationship of Dk and EWC was absent with the addition of SNPs. Therefore, the result of this study proposed the new approach to solve the frequent trouble of hydrogel lenses.

### 3.7. Mechanical Properties

Generally, lower modulus lenses can provide more comfort and fit for wearers [6,22]. Figure 7 shows that Young’s modulus of SNPs-loaded hydrogel increased almost linearly with the SNP content. The Young’s modulus of lenses increased from 0.41 MPa to 1.02 MPa with increasing SNPs ratio (0 wt% for S0 and 1.8 wt% for S5). The reinforcement of the hydrogel suggests that SNPs did serve as the crosslinker for the poly(HEMA-*co*-NVP) molecular chains. It is also reported in the literature that silica nanoparticles can increase the mechanical properties [14].

### 3.8. Protein Deposition

Basically, the deposition of protein has the close relationship with the wettability of hydrogel lenses. Because the wettability was not affected by the addition of SNPs, the deposition of proteins was not affected significantly either, as shown in Table 1. Comparing with 0 wt% of SNPs, the apparent amount of protein adsorption slightly varied around 0.75 and 0.78 nmol/cm^2^ for HSA and between 3.1 and 3.3 nmol/cm^2^ for lysozyme. The high HSA and lysozyme adsorption may be explained based on the properties of hydrogel materials, especially NVP monomers [3,17,23,24,25].

### 3.9. Cytotoxicity Test

**Figure 8:**
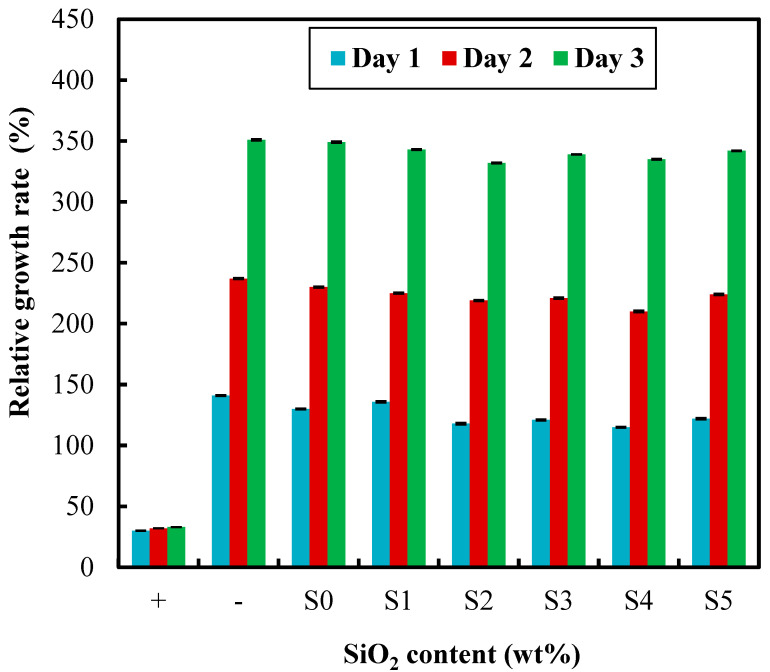
Effect of SNPs content on cytotoxicity.

The biocompatibility of an ophthalmic device, especially contact lenses, is significant factor determined via cellular behavior [6,21]. The in vitro cytotoxicity test was applied to observe the growth of L929 cells based on sample’s extracted medium. In comparison to positive and negative controls, the L929 cells using extracted medium could growth normally after three days culture as shown in Figure 8. The result determined that the addition of SNPs content in hydrogel lenses did not influence the growth of L929 cells through cytotoxicity test.

### 3.10. Comparison with Commercial Contact Lenses

The Dk and EWC of SNPs hydrogel lenses were comparable or higher than those non-silicone commercial products such as Biomedics XC, Biomedics 38, Acuvue 2, and Acuvue Advance (Table 2) [3,26]. Although the Dk values of these SNP-loaded lenses were lower than those silicone commercial lenses such as Air Optix Night & Day, Air Optix, Acuvue Oasys, and PureVision, the EWC values were higher and the moduli were similar. The results of this research indicated that a small amount of SNPs would confer conventional hydrogel lenses improved ophthalmic properties to non-silicone commercial contact lenses including Biomedics XC and Acuvue Advance. These SNPs lenses can be considered to apply for ophthalmology materials as contact lenses.

## 4. Conclusions

Silica sol was prepared through the hydrolysis of tetraethoxysilane (TEOS) followed by blending with hydroxyethyl methacrylate (HEMA) and N-vinylpyrrolidone (NVP) before photoinitiated polymerization. With the silica content increasing to 1.8 wt%, the oxygen permeability of the resultant hydrogel was increased from 20 barrer to 54 barrer, while the equilibrium water content and contact angle remained around 52% and 60°, respectively. In addition, the Young’s modulus increased from 0.4 MPa to 1 MPa when the content of silica nanoparticles increased to 1.8 wt%.

## Figures and Tables

**Table 1:** Hydrophilicity, oxygen permeability, wettability and protein deposition of SNP-loaded contact lenses.

Sample	SNP Content (wt%)	EWC (%)	Dk (barrer)	Dk/t (barrer/mm)	Contact Angle (°)	Protein Deposition (nmol/cm^2^)	
						HSA	Lysozyme
S0	0	52.5 ± 1.6	20.6 ± 1.0	198 ± 9	60.5 ± 2.3	0.76 ± 0.01	3.10 ± 0.05
S1	0.18	51.5 ± 2.1	26.6 ± 1.2	257 ± 11	60.1 ± 3.1	0.78 ± 0.01	3.21 ± 0.05
S2	0.46	53.5 ± 1.7	36.7 ± 1.5	355 ± 19	61.1 ± 2.9	0.75 ± 0.01	3.27 ± 0.04
S3	0.92	52.9 ± 1.9	42.8 ± 1.7	414 ± 11	59.5 ± 4.1	0.76 ± 0.01	3.16 ± 0.05
S4	1.37	51.9 ± 2.0	50.3 ± 1.9	494 ± 13	60.1 ± 3.3	0.75 ± 0.01	3.23 ± 0.06
S5	1.82	53.1 ± 1.8	54.3 ± 2.2	534 ± 15	61.2 ± 4.5	0.76 ± 0.01	3.29 ± 0.05

**Table 2:** Properties comparison of this study and commercial lenses.

Product	Manufacturer	Dk (barrer)	EWC (%)	Contact angle (°)	Modulus (MPa)	Principle Monomers
Air Optix Night & Day	CIBA Vision	140	24		1.52	DMA, TRIS, siloxane monomer
Air Optix		110	33	44.4	1.00	DMA, TRIS, siloxane monomer
Acuvue Oasys	Johnson & Johnson Vision Care	103	38	78.7	0.72	MPDMS, DMA, HEMA, siloxane macromer, TEGDMA, PVP
Acuvue Advance		60	47	65.6	0.43	MPDMS, DMA, HEMA, EGDMA, siloxane macromer, PVP
Acuvue 2		19	58			HEMA, MAA, EGDMA
Pure Vision	Bausch & Lomb	91	36	93.6	1.10	TEGDMA, NVP, TPVC, NCVE, PBVC
Biomedics XC	CooperVision	44	60			HEMA, MAA, PC, TEGDMA
Biomedics 38		8.4	38	30	0.81	HEMA, EGDMA
S4	This work	50.3	51.9	60.1	0.88	HEMA, NVP, SNPs
S5		54.3	53.1	61.2	1.02	

^PVP^ polyvinyl pyrrolidone; ^MPDMS^ monofunctional polydimethylsiloxane; ^DMA^ N,M-dimethylacrylamide; TEGDMA tetraethyleneglycol dimethacrylate; ^TRIS^ trimethyl siloxysilyl; ^TPVC^ tris-(trimethyl siloxysilyl)propylvinyl carbamate; ^NCVE^ N-carboxyvinyl ester; ^PBVC^ poly-(dimethysiloxy) di-(silylbutanol) bis-(vinyl carbamate).
